# Supplementary material for: Polar Lipids in Starch-Rich Commodities to be Analyzed with LC-MS-Based Metabolomics—Optimization of Ionization Parameters and High-Throughput Extraction Protocols
Source: Metabolites. 2019 Aug 12;9(8):167. doi: 10.3390/metabo9080167 (PMC6724080; doi:10.3390/metabo9080167)
Supplement: Supplementary file 1 [file metabolites-09-00167-s001.zip › SupplementaryMaterial_Claassen.pdf]

metabolites

MDPI

Article

Polar lipids in starch-rich commodities to be analyzed with LC-MS-based metabolomics – Optimization of ionization parameters and high-throughput extraction protocols

Christin Claassen [1,2], Jürgen Kuballa [1,*] and Sascha Rohn [2]

[1] GALAB Laboratories GmbH, Research and Development, Am Schleusengraben 7, 21029 Hamburg, Germany; christin.claassen@galab.de
[2] Hamburg School of Food Science, Institute of Food Chemistry, University of Hamburg, Grindelallee 117, 20146 Hamburg, Germany; rohn@chemie.uni-hamburg.de
* Correspondence: juergen.kuballa@galab.de; Tel.: +49-40-368077-410

Received: 27 June 2019; Accepted: 8 August 2019; Published: date

Table S1. Composition of standard solutions obtained from Matreya LLC.

**Acylated steryl glucosides (based on campesterol, sitosterol, stigmasterol)**		**Galactocerebrosides**		**Glucocerebrosides**	
C16:0	34%	C18:0(OH)	36%	C14:0(OH)	trace
C18:0	8%	C20:0(OH)	1%	C16:0(OH)	79%
C18:1	8%	C22:0(OH)	8%	C18:0(OH)	trace
C18:2	36%	C23:0(OH)	6%	C22:0(OH)	8%
C18:3	4%	C24:0(OH)	25%	C23:0(OH)	1%
C20:0	1%	C24:1(OH)	9%	C24:0(OH)	9%
C22:0	4%	C25:0(OH)	4%		
C23:0	2%	C25:1(OH)	2%		
C24:0	2%	C26:0(OH)	2%		
		C26:1(OH)	2%		

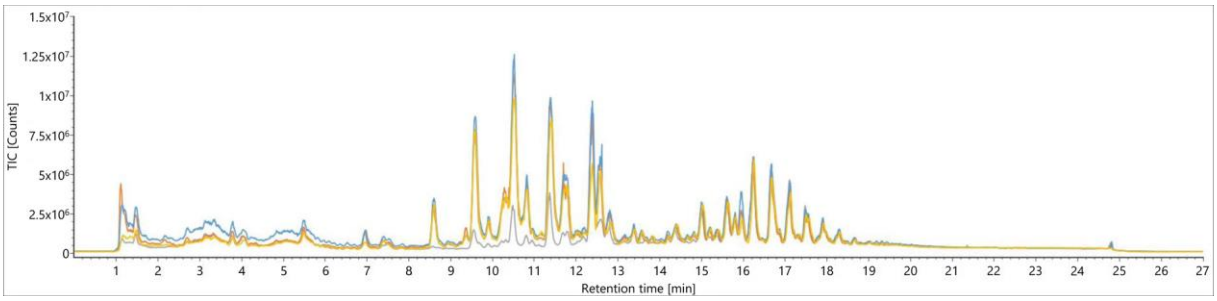

Figure S1. Total ion chromatograms of four QC samples consisting of extracts according to Bligh and Dyer (blue) [1], Folch (orange) [2], Reis using *n*-hexane (grey) [3], and Matyash (yellow) [4].

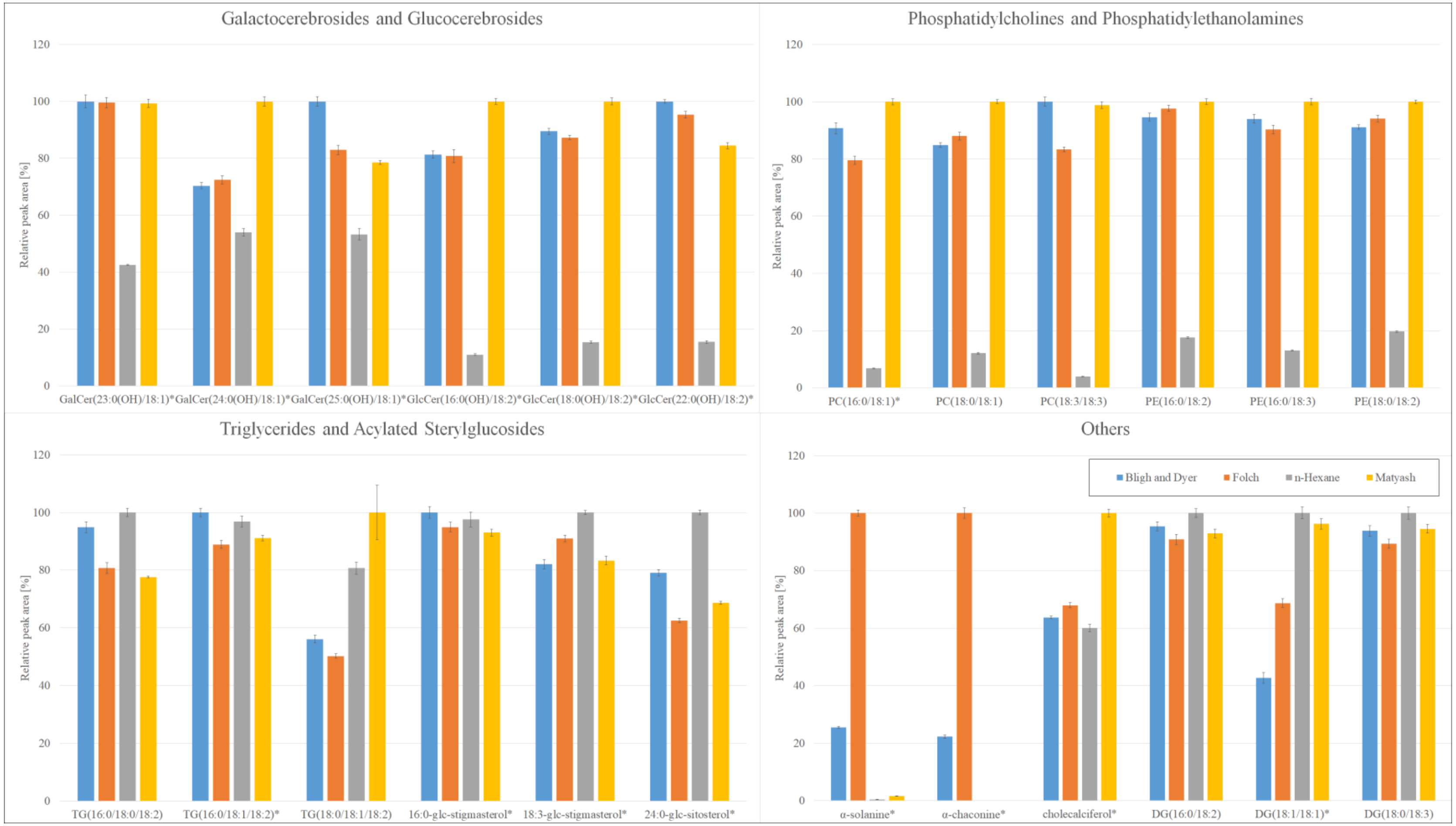

Figure S2. Comparison of four extraction protocols according to Bligh and Dyer (blue),[1] Folch (orange) [2], Reis using *n*-hexane (grey) [3], and Matyash (yellow)[4] on the basis of relative peak areas of various lipophilic potato metabolites in the respective QC-samples. Metabolites marked with asterisks were identified and proven with reference standards.

metabolites MDPI

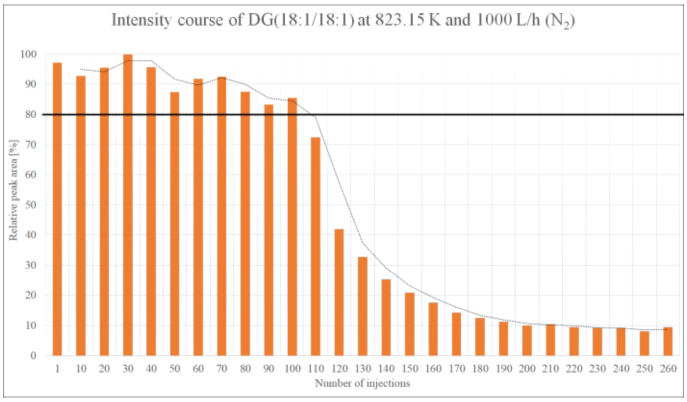

Figure S3. Relative peak areas of the diglyceride DG(18:1/18:1) in chloroform-based extracts [2] during an analysis time of 5 days using a desolvation temperature of 823.15 K and desolvation gas flow of 1000 L/h (N_2).

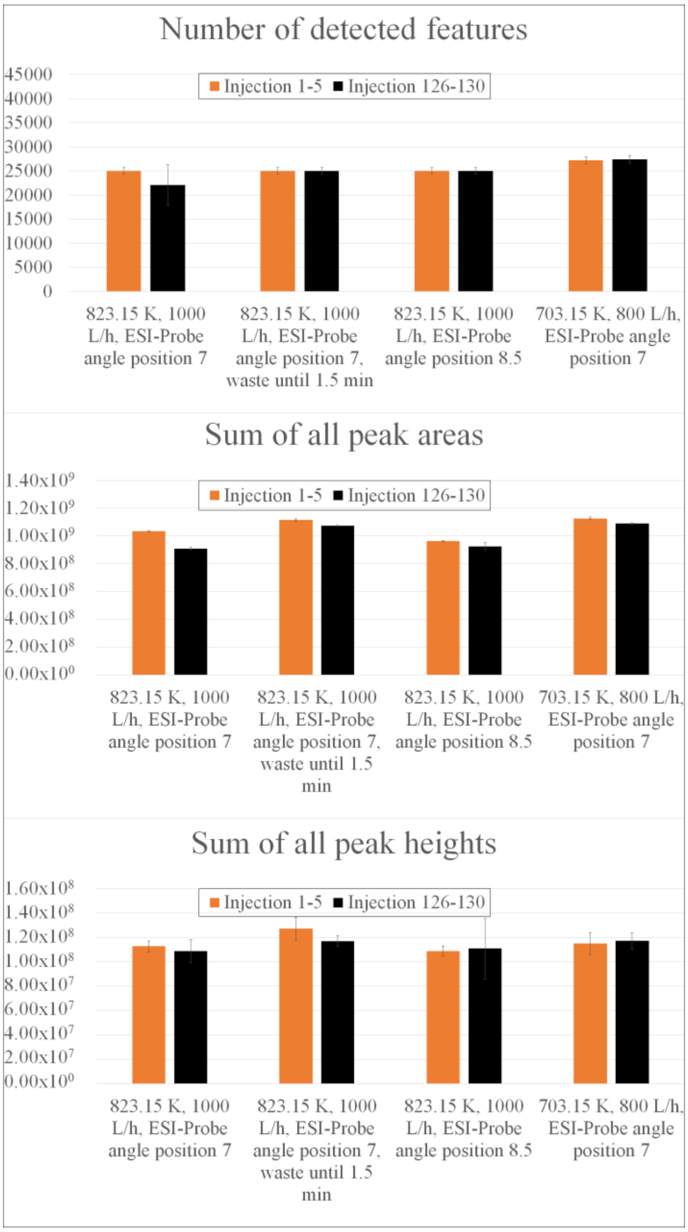

Figure S4. Comparison of ionization parameters varying desolvation settings, removing injection peaks, and increasing instrumental angles and distances with regard to the number of detected mass features, the sum of all peak areas, and the sum of all peak heights.

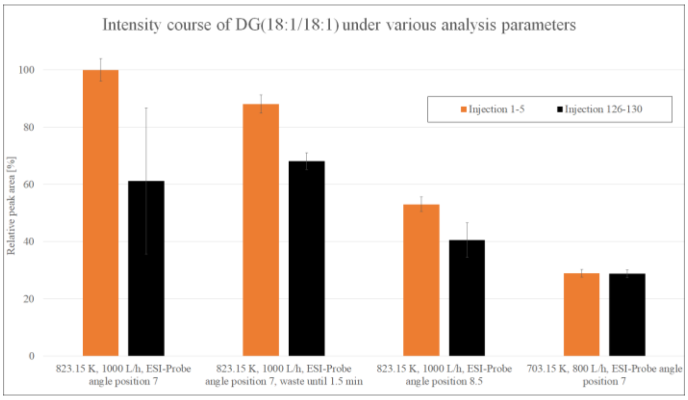

Figure S5. Relative peak areas of the diglyceride DG(18:1/18:1) in chloroform-based extracts during an analysis time of 2.5 days using different analysis parameter (desolvation temperature and gas flow, removal of injection peak, ESI-Probe angles).

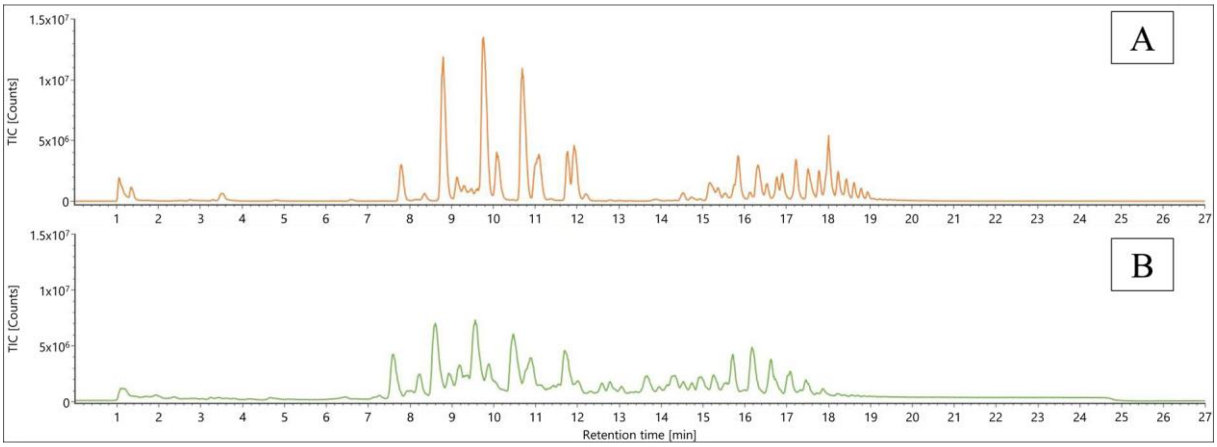

Figure S6. Total ion chromatograms of two QC samples consisting of extracts: (**A**) according to Folch (orange) [2] and (**B**) a “green” Folch approach (green) [5].

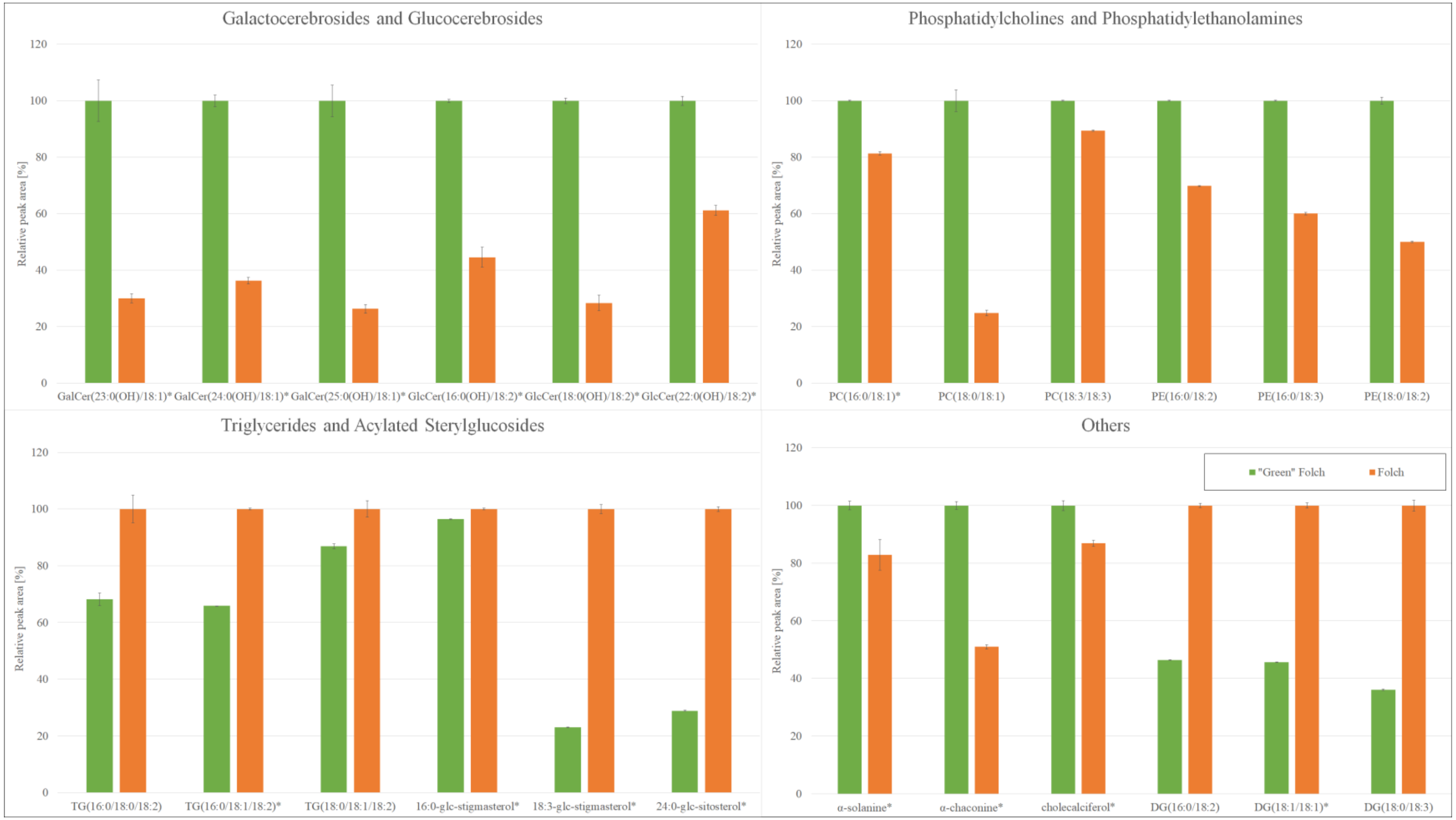

Figure S7. Comparison of the extraction according to Folch (orange) [2] *vs.* a "green" Folch approach (green) [5] on the basis of relative peak areas of various lipophilic potato metabolites in the respective QC-samples. Metabolites marked with asterisks were identified and proven with reference standards.

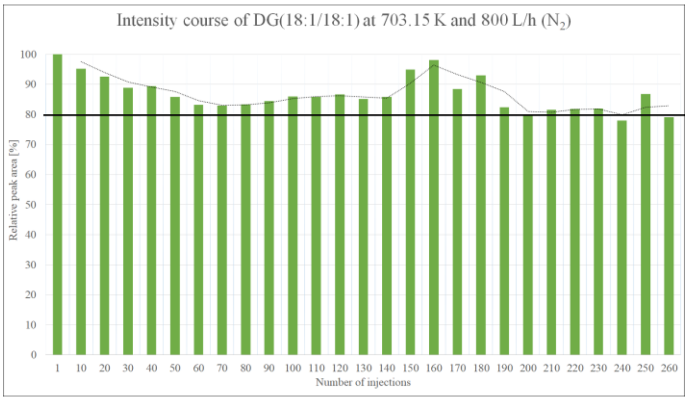

Figure S8. Relative peak areas of the diglyceride DG(18:1/18:1) in ethyl acetate-based extracts [5] during an analysis time of 5 days using a source temperature of 703.15 K and a desolvation gas flow of 800 L/h (N_2).

References

1. Bligh, E.G.; Dyer, W.J. A rapid method of total lipid extraction and purification. *Can. J. Biochem. Physiol.* **1959,** *37*, pp. 911-917. https://doi.org/10.1139/y59-099.
2. Folch, J.; Lees, M.; Sloane Stanley, G.H. A simple method for the isolation and purification of total lipides from animal tissues. *J. Biol. Chem.* **1957,** *226*, pp. 497-509.
3. Reis, A.; Rudnitskaya, A.; Blackburn, G.J.; Mohd Fauzi, N.; Pitt, A.R.; Spickett, C.M. A comparison of five lipid extraction solvent systems for lipidomic studies of human LDL. *J. Lipid Res.* **2013,** *54*, pp. 1812-1824. https://doi.org/10.1194/jlr.M034330.
4. Matyash, V.; Liebisch, G.; Kurzchalia, T.V.; Shevchenko, A.; Schwudke, D. Lipid extraction by methyl-tert-butyl ether for high-throughput lipidomics. *J. Lipid Res.* **2008,** *49*, pp. 1137-1146. https://doi.org/10.1194/jlr.D700041-JLR200.
5. Breil, C.; Abert Vian, M.; Zemb, T.; Kunz, W.; Chemat, F. "Bligh and Dyer" and Folch Methods for Solid-Liquid-Liquid Extraction of Lipids from Microorganisms. Comprehension of Solvatation Mechanisms and towards Substitution with Alternative Solvents. *Int. J. Mol. Sci.* **2017,** *18*, pp. 708. https://doi.org/10.3390/ijms18040708.

© 2019 by the authors. Submitted for possible open access publication under the terms and conditions of the Creative Commons Attribution (CC BY) license (http://creativecommons.org/licenses/by/4.0/).
